# Epigenetic Aging and Treatment Response to Semaglutide in the SLIM LIVER Study

**DOI:** 10.21203/rs.3.rs-7697256/v1

**Published:** 2025-10-01

**Authors:** Michael Corley, Alina Pang, Douglas Kitch, Amy Kantor, Fred Sattler, Pablo Belaunzaran-Zamudio, Todd Brown, Alan Landay, Jordan Lake, Kristine Erlandson

**Affiliations:** University of California, San Diego; University of California, San Diego; Harvard T.H. Chan School of Public Health; Harvard T.H. Chan School of Public Health; University of Southern California Keck School of Medicine; National Institute of Allergy and Infectious Diseases (contractor); John Hopkins University School of Medicine; University of Texas Medical Branch; UTHealth Houston; University of Colorado- Anschutz Medical Center

**Keywords:** Epigenetics, HIV, Aging, Semaglutide, GLP-1, DNA methylation, Epigenetic clock, geroscience, liver, MASLD

## Abstract

**Background:**

Semaglutide, a glucagon-like peptide-1 receptor agonist (GLP-1 RA), improves metabolic health and reduces liver fat in people with HIV (PWH) and metabolic dysfunction-associated steatotic liver disease (MASLD). Whether changes in epigenetic aging biomarkers reflect these clinical benefits remains unknown.

**Methods:**

We conducted a post hoc analysis of the SLIM LIVER study (ACTG A5371), a 24-week, single-arm trial of semaglutide (1.0 mg weekly) in PWH and MASLD. Epigenetic aging was assessed at baseline and 24 weeks using DNA methylation–based epigenetic clocks: DunedinPACE (pace of aging), PCGrimAge (mortality risk), and PCDNAmTL (methylation-derived telomere length). Participants were stratified by change in epigenetic markers (decrease vs. increase); clinical responses were compared across anthropometric, metabolic, and physical function outcomes.

**Results:**

We observed a stable pace of aging was maintained over 24 weeks (n=41) with a median change of DunedinPACE of +0.018 (IQR: –0.023 to +0.053), PCDNAmTL (median –0.006 kb; IQR: –0.073 to +0.054), and PCGrimAge (median +0.54 years; IQR: –0.33 to +1.26). Seventeen (41.5%) showed a decrease in DunedinPACE with significantly greater reductions in liver fat (*p* = 0.024) and improved gait speed (*p* = 0.081), corresponding to a ~0.8 day (minimum, –0.0048) to ~19.5 days (maximum, –0.116) deceleration. Participants with increased PCDNAmTL (n=20) similarly demonstrated significantly greater improvements in gait speed (*p*= 0.012). No significant clinical associations were observed with changes in PCGrimAge.

**Conclusions:**

These findings provide preliminary evidence that semaglutide may modulate epigenetic age biomarkers, with DunedinPACE and PCDNAmTL tracking improvements in hepatic and physical function. Integration of epigenetic biomarkers into future trials may enhance gerotherapeutic precision by identifying individuals most likely to benefit from GLP-1RA therapy and by enabling minimally invasive monitoring of biological aging.

**Trial Registration:**

ClinicalTrials.gov ID: NCT04216589

## INTRODUCTION

Glucagon-like peptide-1 receptor agonists (GLP-1 RAs), such as semaglutide, have emerged as a transformative class of therapeutics that promote weight loss, improve glycemic control, and provide systemic benefits across multiple organ systems^1–4^. Beyond their established role in diabetes management, GLP-1 RAs have demonstrated cardiovascular protection, potential neuroprotective effects, and improvement in inflammation and endothelial function, positioning them as promising therapeutic agents for the geroscience field^1–3,5,6^. The properties of GLP-1 RAs are particularly relevant for metabolic dysfunction–associated steatotic liver disease (MASLD), a leading comorbidity in people with HIV (PWH) characterized by excess intrahepatic triglyceride (IHTG) accumulation, insulin resistance, oxidative stress, and systemic inflammation^7^. While lifestyle-induced weight loss remains the cornerstone of MASLD management^8^, semaglutide^9^ has shown efficacy in reducing hepatic steatosis and improving broader metabolic parameters and physical function^10,11^. Such benefits may be especially important for PWH, who frequently experience an accelerated aging phenotype driven by chronic immune activation and metabolic dysregulation^12^.

Recent advances in epigenetic biomarker research have enabled the quantification of biological aging through DNA methylation-based “epigenetic clocks”^13^. These clocks serve as minimally invasive proxies of biological aging and have been linked to metabolic traits such as body mass index, visceral adiposity, insulin resistance, and liver disease severity^14–16^. In PWH, epigenetic age acceleration (EAA) is frequently observed, with estimates ranging from 3 to 7 years above chronological age^17–19^. Recent evidence suggests that EAA may also reflect liver disease progression. In a study of 325 individuals with MASLD, advanced fibrosis was associated with a 5% faster pace of aging, as measured by DunedinPACE, and a 10% reduction in telomere length, captured by DNAmTL, compared to those without fibrosis^16^. Similarly, in a separate study of individuals with biopsy-confirmed non-alcoholic steatohepatitis (NASH), EAA measured by the Horvath clock correlated with hepatic collagen content, though not fibrosis stage, and revealed differentially methylated CpG sites enriched in developmental and transcriptional regulatory pathways^15^. These findings underscore the potential of epigenetic aging measures as biomarkers of liver disease severity and therapeutic responsiveness. However, it remains unclear whether longitudinal changes in epigenetic biomarkers track with clinical improvements in response to interventions such as GLP-1 RA therapy.

To address this, we conducted a post hoc epigenetic analysis of participants from the SLIM LIVER study (Advancing Clinical Therapeutics Globally for HIV/AIDS and Other Infections (ACTG) A5371; NCT04216589), an open-label, single arm, Phase 2 clinical trial of semaglutide in PWH with MASLD. In the parent study, 24 weeks of low-dose semaglutide (1 mg subcutaneously weekly) significantly reduced IHTG by 31.3%, improved insulin sensitivity (approximately 1.5-unit decrease in Homeostatic Model Assessment of Insulin Resistance (HOMA-IR)), and lowered triglyceride levels by 27 mg/dL^11^. A secondary analysis also revealed preserved or improved physical function, with a significant reduction in the prevalence of slow gait speed (< 1 m/s) despite modest muscle loss^10^. We hypothesized that within-individual changes in epigenetic aging biomarkers DunedinPACE, PCGrimAge, and DNAm telomere length (PCDNAmTL) over 24 weeks of semaglutide treatment would be associated with improvements in hepatic fat, metabolic markers, and physical function. This exploratory analysis aimed to assess the extent to which biological aging is modifiable in response to semaglutide and whether such changes are associated with therapeutic benefit in PWH with MASLD.

## 1. METHODS

### 1.1. Trial Population

The SLIM LIVER study ([ACTG] protocol A5371; NCT04216589) was a Phase 2b, single-arm, open-label, 24-week, pilot study designed to evaluate the effect of semaglutide on IHTG and metabolic health among PWH and MASLD^11^. Participants were enrolled from nine ACTG-affiliated clinical research sites between February 2021 and September 2022. Eligible participants were aged ≥ 18 years, living with HIV on stable antiretroviral therapy (ART) with suppressed HIV-1 RNA (< 50 copies/mL), had ≥ 5% IHTG as quantified by magnetic resonance imaging proton-density fat fraction (MRI-PDFF), and demonstrated central adiposity and either insulin resistance (HOMA-IR > 3.0) or pre-diabetes (fasting glucose 100–125 mg/dL or hemoglobin A1c (HbA1c ) 5.7–6.4%). Exclusion criteria included previous GLP-1 RA use within 24 weeks, diabetes mellitus, significant alcohol use, and other causes of liver disease. Physical function was measured at baseline and week 24 by assessing the time to rise from a chair 5 and 10 times and 4-meter gait speed, where gait speed was calculated as the average of 2 measurements at usual pace. Slow gait speed was defined as walking < 1 m/sec. Each site obtained institutional review board approval, and all participants provided written informed consent.

### 1.2. SLIM LIVER Epigenetic Sub-study Participant Selection

We evaluated the longitudinal changes in epigenetic age estimates at two time points, at baseline and after 24 weeks of low-dose semaglutide, for 41 participants enrolled with available peripheral blood mononuclear cells (PBMCs).

### 1.3. DNA Methylation Profiling and Epigenetic Age

DNA was isolated from PBMCs using a Zymo Research Quick-DNA microprep kit. 500 ng of DNA was treated with bisulfite using the EZ DNA Methylation kit from Zymo Research, following the manufacturer’s instructions. The bisulfite-treated DNA samples were randomly assigned to a well on the Infinium HumanMethylationEPIC BeadChip, which was then amplified, hybridized, stained, washed, and imaged with the Illumina iScan SQ instrument to obtain raw image intensities. To pre-process the DNA methylation data, we used the *minfi* pipeline^20^, and low quality samples were identified using the *qcfilter()* function from the ENmix package^21^, using default parameters. A total of 82 samples (41 baseline and 41 follow up), representing 100% of the original samples, passed the quality assurance and quality control (p < 0.05) and were deemed to be high quality samples. Our focus was on the second-generation principal component-derived epigenetic clock, PCGrimAge^22,23^, a measure of biological aging that incorporates DNA methylation-based estimates of biomarkers associated with age-related mortality risk, the third-generation clock, DunedinPACE^24^, a measure of the pace of aging crucial for understanding the impact of interventions on epigenetic aging, and Lu’s telomere length predictor based on 140 CpGs ^25^. Epigenetic clocks were calculated according to published methods^19^ from processed DNA methylation data. To enhance the reliability of GrimAge and DNAmTL estimates, we utilized its principal-component versions using the custom R script available via GitHub (https://github.com/MorganLevineLab/PC-Clocks)^23^. The pace of aging clock, DunedinPACE, was calculated using the *PACEProjector* function from the DunedinPACE package available via GitHub (https://github.com/danbelsky/DunedinPACE).

### 1.4. Statistical Analysis

This post hoc analysis assessed whether changes in epigenetic aging markers over 24 weeks of semaglutide treatment were associated with differential responses across hepatic, metabolic, and physical function domains. Descriptive statistics were reported as medians with interquartile ranges (IQRs) for continuous variables and frequencies with percentages for categorical variables. Participants were stratified into “decreased” vs. “increased” epigenetic age change groups based on directionality of change in three biomarkers: DunedinPACE, PCGrimAge, and PCDNAmTL. Directionality (increase vs. decrease) was assessed separately for DunedinPACE, PCGrimAge, and PCDNAmTL. Group comparisons for percent changes in clinical measures (e.g., IHTG, HOMA-IR, HbA1c, BMI, physical function) were conducted using the Kruskal-Wallis test. For epigenetic biomarkers, changes from baseline to week 24 were analyzed using Wilcoxon signed-rank tests for within-group comparisons.

## 2. RESULTS

### 3.1. SLIM LIVER Epigenetic Substudy Characteristics.

Characteristics of the overall SLIM LIVER cohort have been previously described^11^. Forty one of 51 enrolled participants had evaluable samples at both time points for our post hoc epigenetic analysis (Table 1). The median age was 52 years (interquartile range [IQR]: 42–58). Obesity and central adiposity were common (by design), with a median body mass index (BMI) of 35 kg/m^2^ (IQR: 31–39) and median waist circumference of 114 cm (IQR: 107–124). All participants were on suppressive ART with HIV-1 RNA levels below 50 copies/mL, and the median CD4 + T-cell count was 701 cells/mm^3^ (IQR: 586–869). Metabolic parameters reflected elevated cardiometabolic risk, with a median HOMA-IR of 3.8 (IQR: 2.8–6.1) and fasting glucose of 98 mg/dL (IQR: 93–107). Median fasting triglycerides were 116 mg/dL (IQR: 95–183), and alanine aminotransferase (ALT) was elevated in 53% of participants. ART regimens were predominantly integrase strand transfer inhibitor (INSTI)-based (82%), with smaller proportions on non-nucleoside reverse transcriptase inhibitor (NNRTI; 22%)- or protease inhibitor (PI; 4%)-based regimens.

### 3.2. Baseline PCGrimAge, DunedinPACE, and PCDNAmTL and changes over 24 weeks of semaglutide

At baseline, PCGrimAge, an epigenetic biomarker trained to predict mortality risk, indicated a biological age of 60.0 years (median; range: 34.3–70.6). The median PCGrimAge acceleration reflecting the difference between epigenetic and chronological age was 7.5 years (IQR: 5.1–9.4), suggesting elevated age-related mortality risk within the cohort for 40 of the 41 participants profiled. The DunedinPACE score, which quantifies the rate of aging (with 1.0 representing the normative pace), had a median value of 0.95 (IQR: 0.88–1.00). Twenty two percent (9 of 41 participants) had a DunedinPACE score calculated at greater than 1.0 at baseline, indicating an accelerated pace of aging for these individuals. PCDNAmTL, a methylation-derived estimate of telomere length, showed a median value of 6.90 units (IQR: 6.80–7.16), reflecting relatively uniform telomere-associated aging across participants. Over 24 weeks of semaglutide, participants maintained a stable pace of aging, with a median DunedinPACE change of + 0.018 (IQR: − 0.023 to + 0.053), stable PCDNAmTL (median − 0.006 kb; IQR: − 0.073 to + 0.054), and minimal change in PCGrimAge (median + 0.54 years; IQR: − 0.33 to + 1.26).

### 3.3. Changes in Epigenetic Age Markers Are Associated with Hepatic and Functional Outcomes following 24 weeks of semaglutide

**Figure 1:**
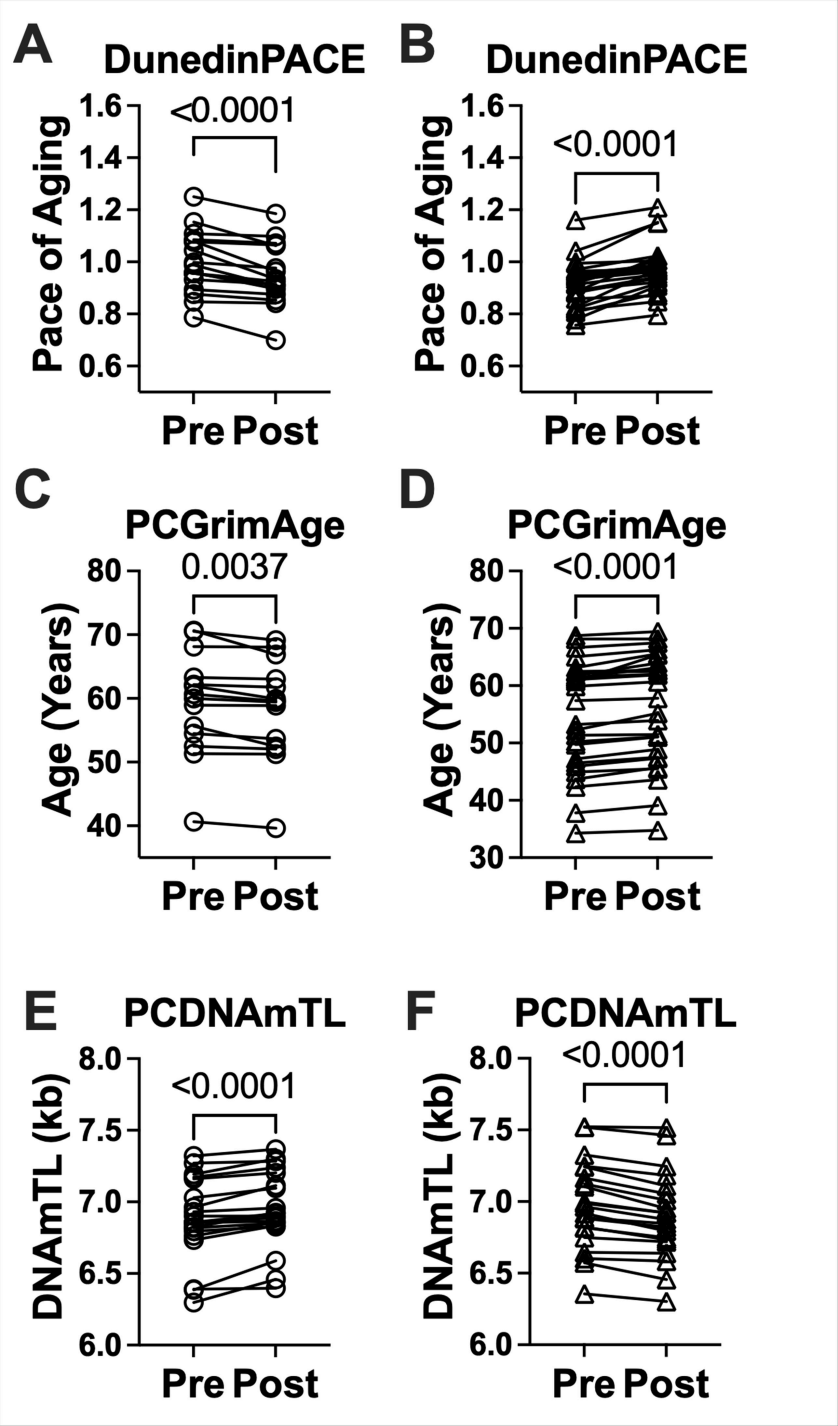
Changes in epigenetic aging biomarkers before and after 24 weeks of semaglutide treatment in people with HIV and MASLD with participant grouping by direction of epigenetic change. (A–B) DunedinPACE, a measure of the pace of aging (1.0 = normative rate), decreased following treatment with 17 participants (41.5%) demonstrating a reduction (panel A) indicating a slower pace of aging and 24 participants demonstrating an increase (panel B). (C–D) PCGrimAge, an epigenetic estimate of biological age and mortality risk, also declined with 14 participants (34.1%) showing reductions in predicted age (panel C) and 27 participants (65.8%) showing an increase (panel D). (E–F) PCDNAmTL, a methylation-derived estimate of telomere length (in kilobases) increased in 20 participants (48.8%) showing predicted telomere elongation (panel E) and 21 (51.2%) participants showing decrease (panel F). Circles indicate participants with beneficial changes (slower pace of aging, reduced epigenetic age, or longer DNAm telomere length), while triangles indicate participants with changes in the opposite direction over the 24-week period. Data are shown as paired values with lines connecting participants.These groupings were used to examine whether directional shifts in epigenetic aging over the 24-week period were associated with differential clinical responses to semaglutide, including outcomes related to anthropometry, metabolic biomarkers, and physical function.

A total of 17 participants (9 male, 8 female; 41.5%) experienced a decrease in DunedinPACE with semaglutide, indicating a slower pace of aging from baseline. 14 participants (8 male, 6 female; 34.1%) demonstrated a decrease in PCGrimAge, indicating a reduction in epigenetic mortality risk, and 20 participants (11 male, 9 female; 48.8%) showed an increase in predicted DNA methylation-based telomere length (PCDNAmTL) (Fig. 1A - F).

**Figure 2:**
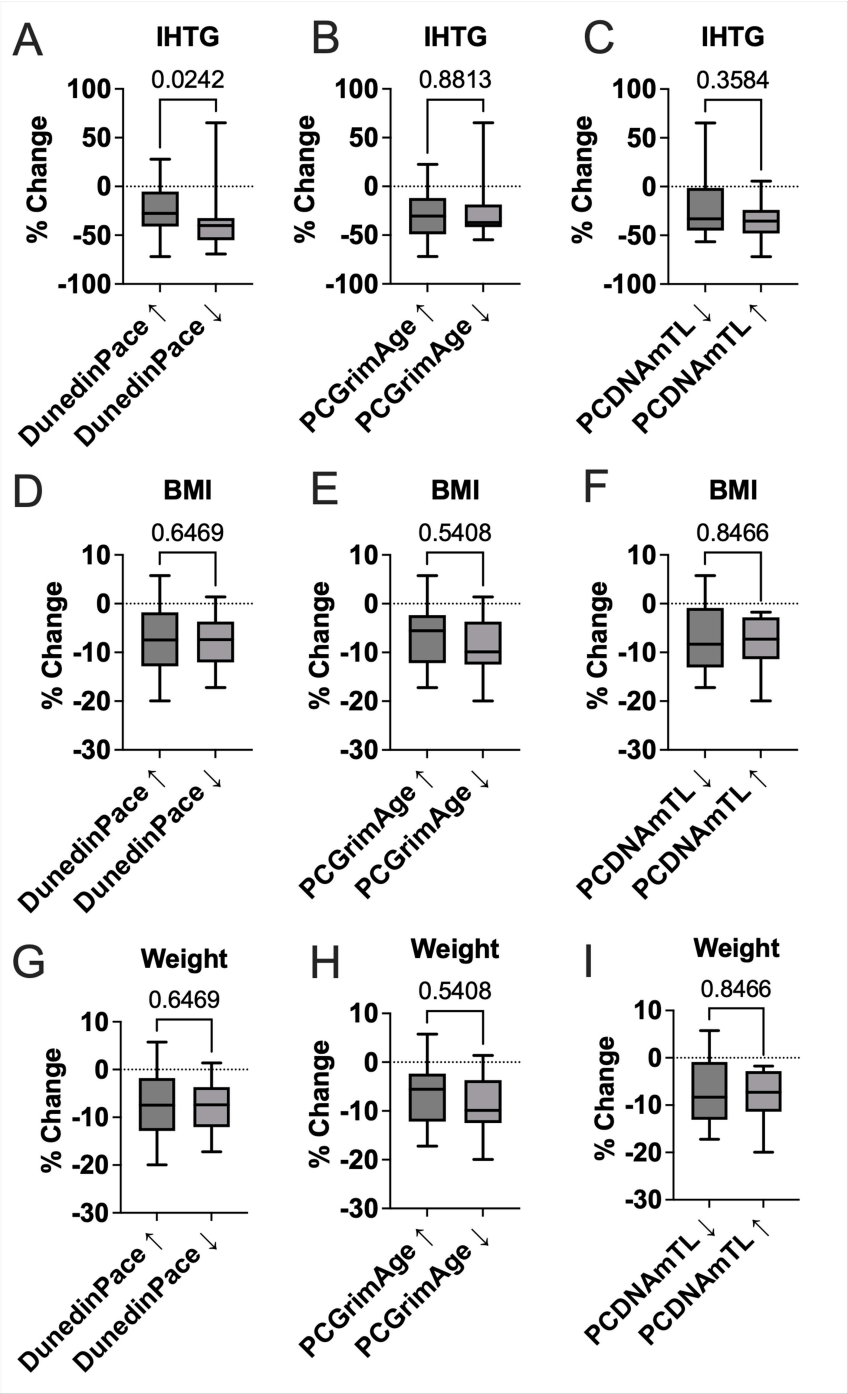
Differences in 24-week changes in IHTG and anthropometric percent following low-dose (1mg) weekly semaglutide by Epigenetic Age Group. Boxplots display the percent change in intrahepatic triglycerides (IHTG; panels A–C), body mass index (BMI; panels D–F), and body weight (panels G–I) stratified by 24 week group changes in three epigenetic aging measures: DunedinPACE, PCGrimAge, and PCDNAmTL. Each panel shows median, interquartile range, and range of percent changes. Statistical comparisons were conducted using the Mann-Whitney nonparametric test, with p-values annotated above each panel.

We examined whether changes in epigenetic aging over the 24 weeks were associated with differential responses to semaglutide treatment across key clinical domains, including anthropometry, metabolic biomarkers, and physical function. Participants were grouped based on direction of change (Increased vs. Decreased) for DunedinPACE, PCGrimAge, and PCDNAmTL. Participants with a decrease in DunedinPACE showed a significantly greater percent reduction in IHTG (*p* = 0.024) compared to those with increased DunedinPACE (Fig. 2). No significant differences were observed for BMI (*p* = 0.63) or weight (*p* = 0.63) (Fig. 2). When stratified by PCGrimAge or PCDNAmTL change groups, no significant group differences were observed for any anthropometric outcome (all *p* > 0.35), including IHTG (*p* = 0.88 for PCGrimAge, *p* = 0.36 for PCDNAmTL), suggesting this association may be specific to DunedinPACE (Fig. 2).

**Figure 3:**
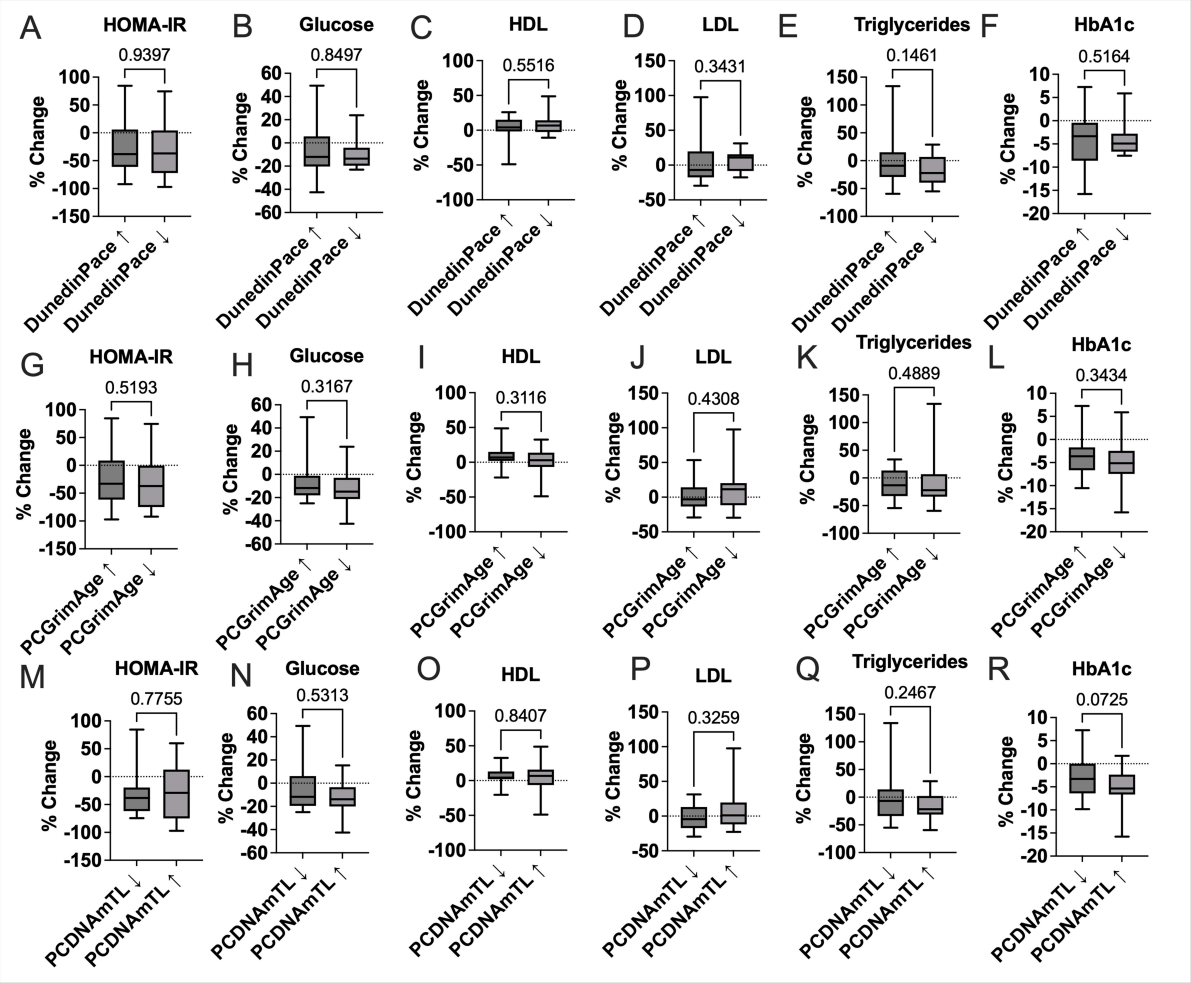
Group differences in lipids and glucose homeostasis percent changes following semaglutide by epigenetic age change. Boxplots display the percent change in insulin resistance (HOMA-IR; panels A, G, M), glucose (panels B, H, N), HDL cholesterol (panels C, I, O), LDL cholesterol (panels D, J, P), triglycerides (panels E, K, Q), and HbA1c (panels F, L, R), stratified by 24-week group changes in DunedinPACE (panels A–F), PCGrimAge (panels G–L), and PCDNAmTL (panels M–R). Each panel shows median, interquartile range, and range of percent changes. Statistical comparisons were conducted using the Mann–Whitney nonparametric test, with *p*-values annotated above each panel.

There were no statistically significant differences in metabolic biomarkers by change group for DunedinPACE, PCGrimAge, or PCDNAmTL. This included HOMA-IR (*p* = 0.94 for DunedinPACE, *p* = 0.78 for PCDNAmTL), fasting glucose, triglycerides, high-density lipoprotein (HDL), and low-density lipoprotein LDL (all *p* > 0.14), indicating that semaglutide-induced improvements in these markers occurred broadly and were not contingent on three epigenetic age dynamics assessed (Fig. 3). However, a trend toward greater reduction in HbA1c was observed among participants with increased PCDNAmTL (*p* = 0.072), suggesting a possible relationship between telomere attrition and glycemic improvement (Fig. 3).

**Figure 4:**
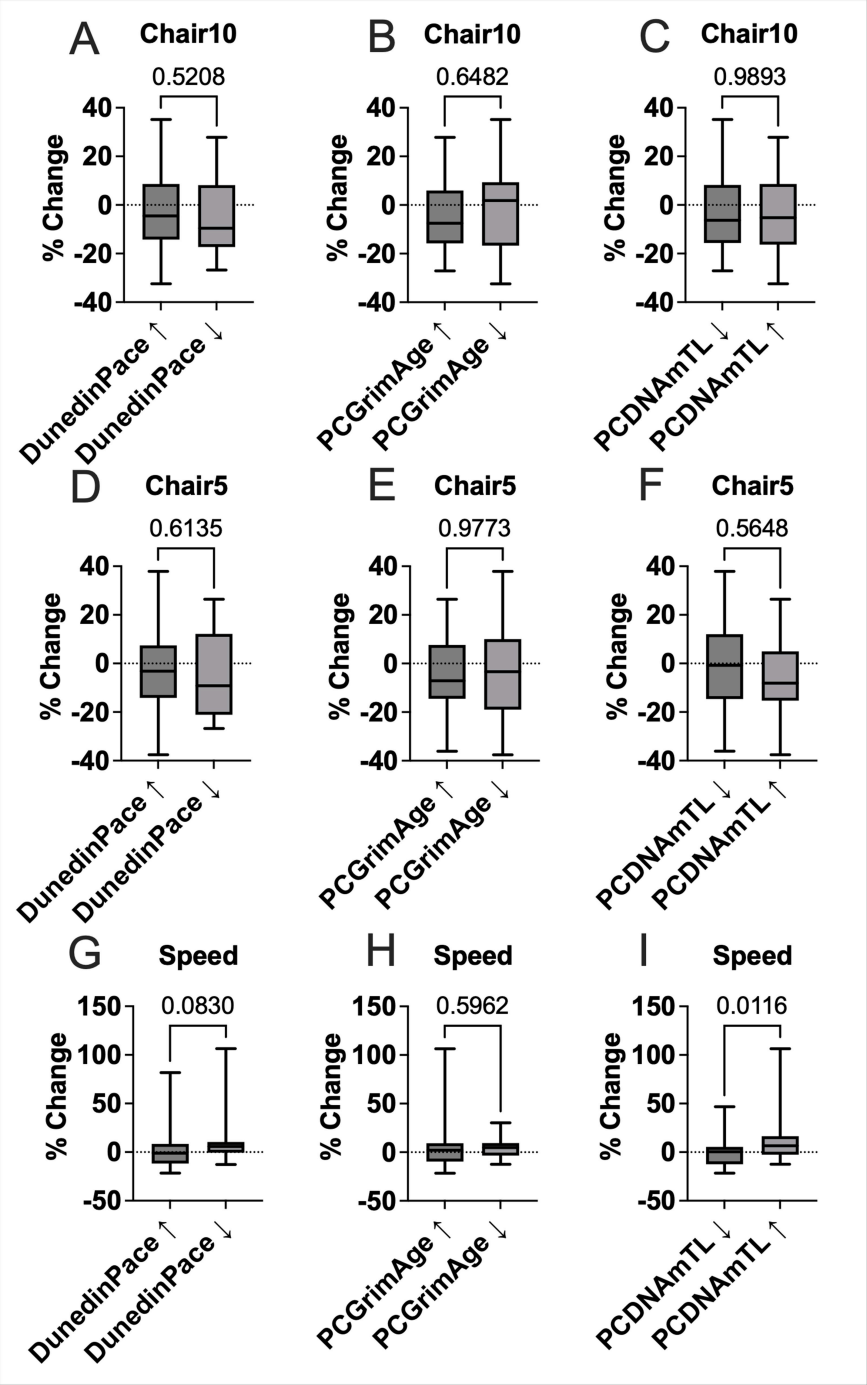
Group differences in physical function percent changes following semaglutide by epigenetic age change. Boxplots display the percent change in physical performance outcomes, including time to complete 10 chair stands (Chair10; panels A–C), time to complete 5 chair stands (Chair5; panels D–F) (% increase indicates slower time to complete), and walking speed (panels G–I) (% increase indicates faster time to complete), stratified by 24-week group changes in three epigenetic aging measures: DunedinPACE, PCGrimAge, and PCDNAmTL. Each panel shows median, interquartile range, and range of percent changes. Statistical comparisons were conducted using the Mann–Whitney nonparametric test, with *p*-values annotated above each panel.

For physical function, no significant differences were found in 5-time or 10-time chair rise time by DunedinPACE, PCGrimAge or PCDNAmTL group (all *p* > 0.50) (Fig. 4). A non-significant trend toward improved gait speed was observed among those with decreased DunedinPACE (*p* = 0.083) and a significant improvement in gait speed was observed among participants with increased PCDNAmTL (*p* = 0.012) (Fig. 4), suggesting that preservation or elongation of telomere length may be linked to better maintenance of physical function following semaglutide treatment.

## 3. DISCUSSION

In this pilot post hoc epigenetic analysis of the SLIM LIVER trial, we evaluated whether changes in epigenetic aging biomarkers, specifically DunedinPACE, PCGrimAge and PCDNAmTL, were associated with anthropometric, metabolic, and physical function changes over 24 weeks of semaglutide treatment in PWH and MASLD. Our findings suggest that a less accelerated pace of aging, as captured by DunedinPACE, may be selectively associated with greater liver fat reduction following semaglutide treatment. While PCGrimAge change was not linked to anthropometric or metabolic outcomes, an increase in telomere length (PCDNAmTL) was significantly associated with improved gait speed, indicating that epigenetic telomere preservation may parallel enhancements in physical function. Conversely, participants with increased PCDNAmTL trended toward greater reductions in HbA1c, hinting at a possible link between semaglutide glycemic improvement and telomere biology. These findings suggest a potential relationship between semaglutide-related changes in specific epigenetic aging biomarkers and improvements in liver fat and physical function. These results merit further investigation of this association in larger cohorts and independent studies to validate the utility of epigenetic age biomarkers as indicators of therapeutic response in GLP-1RA therapy.

The observed reduction in liver fat among select individuals with slowing DunedinPACE may reflect improved metabolic flexibility and hepatic lipid mobilization in reponse to semaglutide. DunedinPACE, is a third-generation DNA methylation-based biomarker that quantifies the pace of biological aging by integrating longitudinal physiological, cellular, and molecular data across multiple organ systems^24^. Unlike first generation epigenetic clocks, it captures short-term changes in systemic function and has been shown to respond to behavioral and pharmacologic interventions^26,27^. Our findings are consistent with prior work demonstrating associations between accelerated DunedinPACE and liver fibrosis severity, insulin resistance, and cardiometabolic risk^16^. The lack of similar associations with body weight or BMI following semaglutide treatment in this study suggests that DunedinPACE may be more sensitive to underlying shifts in tissue-specific metabolic health such as intrahepatic lipid dynamics than to gross anthropometric changes alone. These findings support further exploration of DunedinPACE as a potential biomarker for semaglutide-related improvements in metabolic aging beyond weight loss alone.

Interestingly, we did not observe significant group differences in glycemic or lipid parameters when stratified by epigenetic age change following semaglutide. Improvements in HOMA-IR, fasting glucose, and triglycerides were seen broadly across the cohort following semaglutide^11^, regardless of whether DunedinPACE or PCGrimAge declined over the study period. These findings suggest that semaglutide’s core metabolic benefits may operate, at least in part, through mechanisms independent of its impact on the three DNA methylation-based epigenetic measures assessed. Alternatively, the lack of association may reflect limited power to detect domain-specific effects given the modest sample size. Importantly, while semaglutide has demonstrated robust metabolic efficacy, its potential as a multi-system gerotherapeutic is only beginning to be explored^28^. Emerging data suggest that GLP-1 RAs may influence aging biology in other organ systems such as the brain, cardiovascular system, and kidneys via effects on inflammation, oxidative stress, and cellular senescence^29^. The SLIM LIVER study provides supporting preliminary evidence that semaglutide may also modulate biological aging trajectories in the liver and musculoskeletal system, as reflected by links to liver fat reduction and gait speed improvement. Future work incorporating broader multi-organ epigenetic and transcriptomic profiling may clarify how GLP-1 RAs influence systemic aging biology beyond metabolic control alone.

We also observed a modest trend toward improved walking speed among participants who exhibited a reduction in DunedinPACE. Although this association did not reach statistical significance, the directionality is biologically plausible and consistent with prior studies linking accelerated epigenetic aging to frailty, slower gait speed, and functional decline in older adults^30,31^. The absence of group differences in chair rise times may be due to skeletal muscle effects, task variability, or limited power. Taken together, these findings suggest that semaglutide-associated slowing of biological aging may contribute to preserved or enhanced mobility. Future studies in borader populations, more granular functional assessments, and extended follow-up durations are needed to fully characterize the relationship between changes in epigenetic aging markers and physical performance trajectories.

In addition to DunedinPACE and PCGrimAge, we evaluated changes in methylation-derived telomere length (PCDNAmTL). While changes in PCDNAmTL were not associated with differences in hepatic or anthropometric outcomes, we observed a trend toward greater HbA1c reduction among participants with telomere increases, potentially linking glycemic improvement to telomere biology^32^. In addition, participants with increased PCDNAmTL indicating preserved or elongated telomeres demonstrated a significant improvement in walking speed, suggesting a possible protective effect of telomere maintenance on physical function. In the SLIM LIVER study we found the prevalence of slow gait speed (< 1 m/sec) decreased from 63% to 46% (P = .029)^10^. These findings align with prior evidence linking longer telomeres to better mobility, mitochondrial integrity, and muscle performance in aging populations^33^. Although exploratory, the directionality of these associations suggests that different dimensions of epigenetic aging may differentially track metabolic versus functional responsiveness to treatment. Larger studies are needed to validate these relationships and clarify whether PCDNAmTL may serve as a prognostic marker for physical resilience in the context of semaglutide.

An important consideration in interpreting these results is the use of a relatively low semaglutide dose (1.0 mg weekly) and a 24-week treatment duration, which may have attenuated the magnitude of clinical effects compared to trials using higher doses over longer periods. For example, phase 3 trials such as the STEP and ESSENCE programs have demonstrated greater reductions in liver fat, weight, and glycemic indices with weekly 2.4 mg dosing over 48 to 72 weeks^1,3,6^. However, for anti-aging applications, prolonged tolerability and safety are critical, and lower, sustained dosing regimens may be more appropriate for long-term use. As such, while higher doses could potentially amplify clinical and epigenetic responses, the current design may reflect a more pragmatic framework for gerotherapeutic implementation. Future studies should explore dose–response effects, compare acute versus chronic trajectories of epigenetic change, and evaluate the durability of biological aging modifications with extended follow-up in target populations, including PWH.

Taken together, our findings provide preliminary evidence that semaglutide may influence biological aging trajectories in a subset of individuals, particularly through deceleration of the pace of aging as measured by DunedinPACE. This slowing was selectively associated with greater reductions in liver fat, suggesting that epigenetic aging dynamics may reflect or contribute to organ-specific treatment responsiveness. Given the disproportionate burden of metabolic dysfunction in PWH and the expanding therapeutic role of GLP-1RA in liver disease, these results highlight the importance of integrating biological aging metrics into future interventional studies. Epigenetic clocks such as DunedinPACE and DNAmTL may serve as minimally invasive biomarkers to stratify risk, monitor longitudinal response, and guide personalized approaches aimed at improving both metabolic and functional health outcomes.

## Figures and Tables

**Table 1:** Baseline Participant Characteristics

Baseline Characteristic	Groups	All participants (N = 41)	IHTG% Change Mean (SD)
Overall			−28.8 (27.3)
Age (years)		52 (41.0, 57.5)	
	<50	18 (44%)	−24.4 (28.5)
	>50	23 (56%)	−32.2 (26.5)
Natal sex	Male	25 (61%)	−24.1 (30.0)
	Female	16 (39%)	−36.2 (21.3)
Race	White	30 (73%)	
	Black	16 (39%)	
	Multiple	1 (2%)	
	American Indian/ Alaska Native	1 (2%)	
	Unknown	3 (7%)	
Ethnicity	Not Hispanic or Latino	31 (61%)	
	Hispanic or Latino	20 (39%)	
HIV-1 RNA (copies/mL)	<20	30 (73%)	−27.3 (27.8)
	<50	11 (27%)	−33.0 (26.9)
CD4 count (cells/mm^3^)		701 (606, 848)	
	<500	5 (12%)	−31.5 (34.5)
	≥500	36 (78%)	−28.4 (26.8)
CD4/CD8 ratio (cells/mm^3^)		1.21 (0.714, 1.72)	
	<1	12 (29%)	−33.1 (26.0)
	≥1	29 (71%)	−27.0 (28.1)


## Data Availability

[The data from this study was submitted to the NCBI Gene Expression Omnibus (GEO) http://www.ncbi.nlm.nih.gov/geo/
